# Three-dimensional endoscopy in lumbar spine surgery as a novel approach for degenerative pathologies: a case report

**DOI:** 10.1093/jscr/rjae540

**Published:** 2024-08-28

**Authors:** Alison Ma, Nathan Xie, Joseph Reidy, Ralph Jasper Mobbs

**Affiliations:** Faculty of Medicine, University of New South Wales, Sydney 2052, Australia; NeuroSpine Surgery Research Group, Sydney, Australia; NeuroSpine Surgery Research Group, Sydney, Australia; Department of Neurosurgery, Prince of Wales Hospital, Sydney 2031, Australia; NeuroSpine Surgery Research Group, Sydney, Australia; Department of Neurosurgery, Prince of Wales Hospital, Sydney 2031, Australia; Faculty of Medicine, University of New South Wales, Sydney 2052, Australia; NeuroSpine Surgery Research Group, Sydney, Australia; Department of Neurosurgery, Prince of Wales Hospital, Sydney 2031, Australia; NeuroSpine Clinic, Prince of Wales Private Hospital, Sydney 2031, Australia

**Keywords:** 3D vision, spine endoscopy, three-dimensional endoscopy, spinal degeneration

## Abstract

Endoscopic spine surgery has evolved exponentially. However, the two-dimensional (2D) view results in lack of stereoscopic vision and depth perception, contributing to the steep learning curve. This case report recounts a world first trial of a three-dimensional (3D) endoscopic system that converts 2D to 3D images and explores its potential role in the surgical management of degenerative lumbar spine diseases. The 3D endoscopic system was used for two patient cases and both 2D and 3D images were displayed side by side and compared. Advantages of the 3D endoscopic system include increased perception of depth, rapid identification of bleeding points, and greater visualization of anatomical details. Field of view and exposure were identical in 2D and 3D views. Limitations include costs and need for additional equipment. Overall, 3D endoscopy improved depth perception, instrument manoeuvrability, and recognition of anatomical details. This case report can guide further research and training in endoscopic spine surgery.

## Introduction

Endoscopic spine surgery has evolved exponentially in the past 30 years [1]. Historically, the main indication for endoscopic spine surgery was lumbar disc herniation. Since then, endoscopes, technology, and optics have improved to target a broader range of indications along the entire spine [2, 3]. However, endoscopic spine surgery has a steep learning curve, and there is also currently no formalized training program for surgeons to learn the technique [4]. One of the main challenges of conventional endoscopic spine surgery is the two-dimensional (2D) view, resulting in a lack of stereoscopic vision and depth perception of the surgical field. Consequently, surgeons are required to acquire the psychomotor skills to work with 2D images [5, 6]. Although surgeons have shown interest in endoscopic spine surgery for skill development and to improve patient recovery, they have also identified that the limited visualization requires ongoing training [7].

More recently, three-dimensional (3D) endoscopes have been used in other fields including otolaryngology surgery and laparoscopic surgery and facilitated improved depth perception, as well as increased precision for surgeons in their early stages of training. However, it was identified that 2D endoscopes are lighter, have better handling, and are more user-friendly, making them more feasible for routine endoscopic approaches compared to 3D endoscopes. In addition, 3D endoscopes have an increased diameter, which is opposite to the philosophy of minimal access in spine endoscopy [8–10].

This case report recounts a world first trial of a 3D endoscopic system that converts 2D images to 3D images using custom algorithms, allowing for a 3D view of the surgical field without the need for a 3D endoscope. The aim of this case report is to explore the role of this new 3D endoscopic system for surgical management of degenerative lumbar spine diseases.

## Case report

### Materials and methods

The 3D endoscopic system was used for two patient cases: (1) radiofrequency ablation (RFA) for facetogenic pain and (2) L4/5 endoscopic decompression for spinal stenosis. Informed consent was obtained from both patients in this case report prior to participation. The Endoscopic Spine system used for both cases included the Uniportal Elliquence (New York) Endoscopic system, a 6.3 mm scope for the RFA, and a 10 mm scope for the spinal stenosis. Additional equipment included a drill, radiofrequency probe, and instruments provided by Matrix Medical Innovations (Sydney, Australia).

Both interventions were performed using standard endoscopic techniques, with the addition of visualization using 3D vision.

### 3D endoscopic system

**Figure 1:**
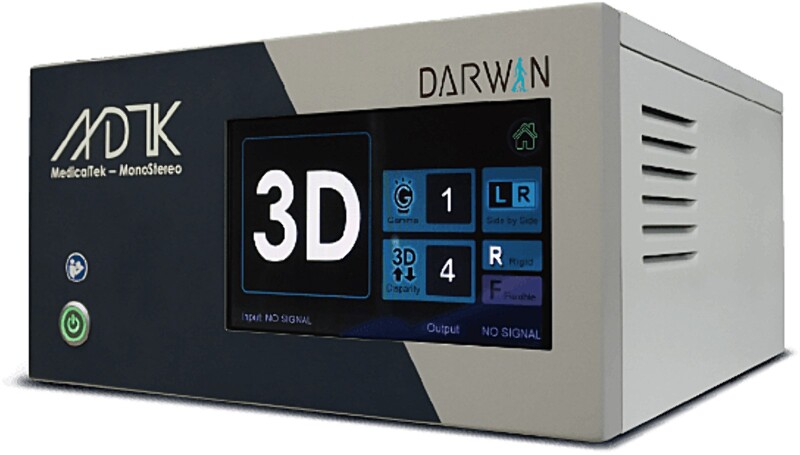
Darwin 3D endoscopic system developed by MedicalTek Co., Ltd.

**Figure 2:**
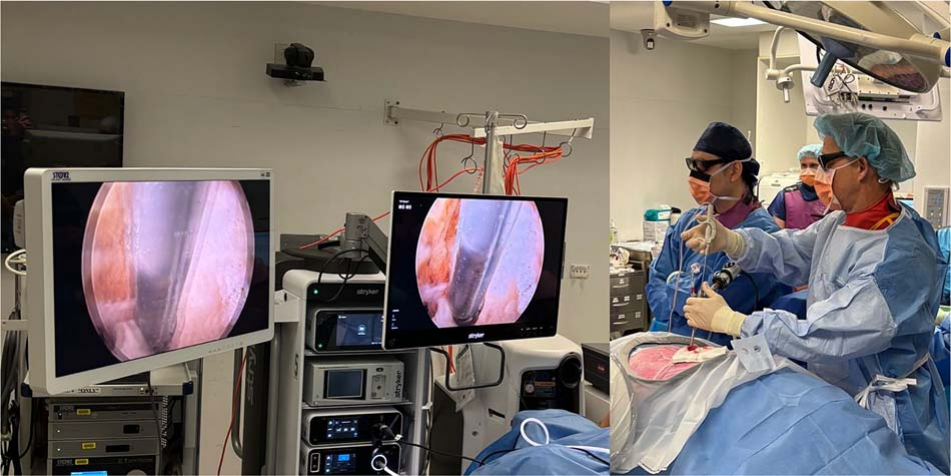
(a) 3D monitor (left) and 2D monitor (right); (b) operating theatre setup and polarization glasses worn to view 3D images.

The Darwin 3D Endoscopic System (Fig. 1) developed by MedicalTek Co., Ltd in Taiwan, was trialled in the surgical procedures for these two patient cases. The system allowed for the use of existing 2D endoscopes, whereby 2D endoscopic images were displayed on a 2D monitor. The 3D endoscopic system used an algorithm that inputs these 2D images and analyses the light reflection and refraction to output 3D images in real time. These 3D images were displayed on a 3D monitor and visualized using polarization glasses. Consequently, both 2D and 3D images were displayed side by side in the operating theatre and were compared (Fig. 2a and b).

## Discussion

This case report explores a world-first trial of a 3D endoscopic system that converts 2D endoscopic images to 3D images in real time, for use in endoscopic spine surgery. Both patients were discharged on the same day without complication.

Subjective advantages of the 3D endoscopic system identified by the senior neurosurgeon (RJM) include increased perception of depth and spatial relationships between structures, as well as rapid identification of bleeding points. Additionally, there was improved navigation and manoeuvrability of instruments including drills and Kerrison punch forceps. Meanwhile, field of view and exposure were identical in both the 2D and 3D views. Advantages identified by the operating theatre staff include improved understanding of the procedure due to greater visualization of anatomical details. However, limitations of the 3D endoscopic system include the additional costs and need for equipment including an additional 3D system and 3D monitor. Another disadvantage is the need to constantly wear polarizing glasses, which darkens the surgical field and may cause dizziness [11].

As the steep learning curve associated with endoscopic spine surgery is a major reason inhibiting widespread implementation, the 3D endoscopic system may be useful for teaching and training, especially during early stages. It may also have the potential to reduce the learning curve associated with endoscopic spine surgery by improving visuospatial orientation [12]. Additionally, this system bridges the gap between 2D and 3D endoscopy as it does not require surgeons who are currently using 2D endoscopes to become familiar with 3D endoscopes, and allows them to maintain comfortability with existing scopes, while benefiting from enhanced depth perception.

## Conclusion

Overall, this case report shows that 3D endoscopy is a valuable learning tool and technique in the surgical management of spine disease by improving depth perception, manoeuvrability of instruments, and recognition of anatomical details. Before widespread implementation, confirmation through case series and analysis of long-term patient outcomes is required to gain a more detailed evaluation of the advantages and limitations of this new technology including accuracy and safety.

## References

[1] Kwon H , Park JY. The role and future of endoscopic spine surgery: a narrative review. Neurospine 2023;20:43–55. 10.14245/ns.2346236.118. 37016853 PMC10080412

[2] Bergamaschi J , Forti F, Bergamaschi E, et al. Development of indications for endoscopic spine surgery: an overview. Int J Transl Med 2023;3:321–33. 10.3390/ijtm3030023.

[3] Kim M , Kim H, Oh SW, et al. Evolution of spinal endoscopic surgery. Neurospine 2019;16:6–14. 10.14245/ns.1836322.161. 31618807 PMC6449828

[4] Maayan O , Mai E, Kim Y, et al. Overview of endoscopic spine surgery and learning curve. Semin Spine Surg 2024;36:101079. 10.1016/j.semss.2024.101079.

[5] Hagan MJ , Remacle T, Leary OP, et al. Navigation techniques in endoscopic spine surgery. Biomed Res Int 2022;2022:1–12. 10.1155/2022/8419739. PMC9444441 36072476

[6] Burkett D , Brooks N. Advances and challenges of endoscopic spine surgery. J Clin Med 2024;13:1439. 10.3390/jcm13051439. 38592293 PMC10932008

[7] Reidy J , Mobbs R. Australian spine surgeon's perspectives on endoscopic spine surgery: an in-depth analysis. Neurospine 2023;20:1321–7. 10.14245/ns.2346912.456. 38171300 PMC10762410

[8] Harada H , Kanaji S, Hasegawa H, et al. The effect on surgical skills of expert surgeons using 3D/HD and 2D/4K resolution monitors in laparoscopic phantom tasks. Surg Endosc 2018;32:4228–34. 10.1007/s00464-018-6169-1. 29603005

[9] Hatzipanayioti A , Bodenstedt S, Bechtolsheim F, et al. Associations between binocular depth perception and performance gains in laparoscopic skill acquisition. Front Hum Neurosci 2021;15:675700. 10.3389/fnhum.2021.675700. 34675789 PMC8524002

[10] Tomazic P , Sommer F, Treccosti A, et al. 3D endoscopy shows enhanced anatomical details and depth perception vs 2D: a multicentre study. Eur Arch Otorhinolaryngol 2021;278:2321–6. 10.1007/s00405-020-06495-6. 33373011 PMC8165070

[11] Heo D , Kim J, Park J, et al. Clinical experiences of 3-dimensional biportal endoscopic spine surgery for lumbar degenerative disease. Oper Neurosurg 2022;22:231–8. 10.1227/ONS.0000000000000090. 35147593

[12] Bickerton R , Nassimizadeh AK, Ahmed S. Three-dimensional endoscopy: the future of nasoendoscopic training. Laryngoscope 2019;129:1280–5. 10.1002/lary.27812. 30628084

